# Effects of visual cues on scene-relative object motion judgments and concurrent heading estimation from optic flow

**DOI:** 10.1167/jov.25.14.20

**Published:** 2025-12-22

**Authors:** Yinghua Yang, Zhoukuidong Shan, Li Li

**Affiliations:** 1 Shanghai Key Laboratory of Brain Functional Genomics, School of Psychology and Cognitive Science, East China Normal University, Shanghai, China; 2 NYU-ECNU Institute of Brain and Cognitive Science at NYU Shanghai, Shanghai, China; 3 Division of Arts and Sciences, New York University Shanghai, Pudong New District, Shanghai, China

**Keywords:** object motion, optic flow, self-movement, heading, causal inference

## Abstract

During self-movement, the visual system uses optic flow to identify scene-relative object motion and estimate the direction of self-movement (heading). Although both processes rely on optic flow, their relationship and the conditions under which independent object motion biases heading estimation remain unclear. The causal inference model predicts that misjudging object motion leads to its integration into heading estimation, causing errors in heading estimation, whereas correct judgments reduce these errors. However, most studies have examined these processes independently. Here we used a dual-task paradigm to investigate how visual cues affect the judgment of scene-relative object motion direction and concurrent heading estimation. Participants viewed a 90° × 90° display simulating self-movement through a three-dimensional cloud with a laterally moving object positioned at 8° or 16° from the simulated heading direction. They judged both the object's motion direction in the scene and their heading direction. Results show that increasing an object’s speed and reducing its positional offset from the simulated heading direction improved the accuracy of scene-relative object motion direction judgment, but did not consistently improve the accuracy of heading estimation. Surprisingly, visual cues such as binocular disparity and object density improved scene-relative object motion direction judgment but reduced heading estimation accuracy. Furthermore, heading errors mostly peaked at object speeds where observers could reliably judge scene-relative object motion direction, challenging the predictions of the causal inference model. These findings provide strong evidence that scene-relative object motion judgment and heading estimation operate independently and question the generality of the causal inference model in explaining heading biases caused by independent object motion.

## Introduction

Navigating complex environments and interacting with surrounding objects are essential for survival. As we move, elements of the environment project a structured pattern of image motion onto our retinae known as optic flow (Gibson, 1947; Gibson, 1950). When we travel along a straight path, optic flow expands and radiates from a point known as the focus of expansion (FoE), which denotes the direction of our self-movement (i.e., heading). The presence of a moving object adds an additional local motion component to the global optic flow, thereby disrupting the global coherent pattern. Previous research found that independent object motion led to small but systematic biases in heading estimation (Layton & Fajen, 2016a; Layton & Fajen, 2016b; Li, Ni, Lappe, Niehorster, & Sun, 2018; Royden & Hildreth, 1996; Warren & Saunders, 1995).

Meanwhile, the perception of scene-relative object motion during self-movement poses another challenge because the retinal motion of the object in this case is a combination of two sources: the object's own motion in the scene and the motion induced by the observer's self-movement. To recover the object's actual motion in the scene, the visual system must discount the self-movement component. One well-studied mechanism for this is flow parsing, in which the global optic flow pattern generated by self-movement is subtracted from the retinal motion of the object (Niehorster & Li, 2017; Rushton, Bradshaw, & Warren, 2007; Rushton & Warren, 2005; Warren & Rushton, 2008; Warren & Rushton, 2009). Flow parsing can, therefore, facilitate scene-relative object motion judgment by reducing the reafferent visual consequences of self-movement. However, flow parsing does not act in isolation, other visual cues (such as the binocular disparity, motion parallax, and object-relative motion signals) as well as non-visual cues (such as vestibular, somatosensory, and proprioceptive information; see Dokka, MacNeilage, DeAngelis, & Angelaki, 2015; Fajen & Matthis, 2013; MacNeilage, Zhang, DeAngelis, & Angelaki, 2012; Xie, Niehorster, Lappe, & Li, 2020) also contribute to scene-relative object motion estimation.

The intertwined challenges for heading estimation and scene-relative object motion judgment during self-movement in the presence of independent object motion raise a central question: Do heading estimation and scene-relative object motion judgments depend on each other or do they operate independently? Some research suggests that they are coupled and both rely on a shared mechanism likely involving the processing of optic flow information. This viewpoint arises from the findings that both heading estimation and flow parsing require sufficient optic flow density (25–35 dots/frame) for robust performance and are similarly influenced by certain statistical properties of optic flow, such as the number of flow vectors and the noise introduced to these vectors (Foulkes, Rushton, & Warren, 2013). However, other research indicates that flow parsing operates independently of heading estimation. This notion is based on findings that, for heading estimation with displays containing superimposed radial and rotational flow fields, the perceived heading direction is biased toward the rotational direction in the flow field (Duffy & Wurtz, 1993; Lappe & Rauschecker, 1995), whereas the scene-relative object motion estimation is consistent with the subtraction of a weighted vector average of radial and rotational flows rather than the global flow pattern based on the shifted heading direction (Warren, Rushton, & Foulkes, 2012). This finding supports the claim that flow parsing does not depend on a prior heading estimate, but instead reflects a direct computation on the optic flow field itself. This claim is further supported by the study by Rushton, Chen, and Li (2018). They compared the precision of heading estimation versus scene-relative object motion judgment under matched display conditions and found that scene-relative object motion judgments were more precise than heading judgments, and changes in depth range or simulated gaze rotation affected the precision of heading estimation but not object motion judgments. These findings show that scene-relative object motion judgments do not depend on prior heading estimation from optic flow.

Recognizing the limitations of these studies that examined heading estimation and scene-relative object motion judgment independently with different visual stimuli, Xing and Saunders (2022) examined these two processes concurrently with the same visual stimuli and found that they exhibited trial-to-trial correlations in performance measurements. Specifically, they found heading errors could predict the accuracy of scene-relative object motion judgments. This finding supports a coupled mechanism for heading estimation and scene-relative object motion judgment. The authors further argued that, in Rushton et al. (2018), object motion was easier to detect because of a small depth range in the visual stimuli that allowed object motion judgments to rely on local deviations from the global optic flow pattern. In contrast, in their study, a relatively large depth range made object motion more difficult to detect, forcing object motion judgments to rely on global flow pattern, which led to significant correlations with heading judgments.

Dokka, Park, Jansen, DeAngelis, and Angelaki (2019) also examined these two processes concurrently with the same visual stimuli. They systematically manipulated the speed of laterally moving objects in the world and found a systematic impact of scene-relative object motion judgment on heading estimation, that is, heading estimation was biased when a moving object was incorrectly judged as stationary. However, the bias significantly decreased when the object was correctly identified as moving in the scene. They thus proposed that the visual system uses causal inference to attribute the sources of retinal motion and simulated their results with a Bayesian causal inference model. Specifically, when the visual system can identify object motion in the scene, it attributes retinal motion to two separate sources: object motion and self-movement. In this case, the system relies primarily on the horizontal component of the self-movement direction for heading estimation, resulting in accurate performance. In contrast, when the visual system misjudges object motion, it attributes the retinal motion to a single source (self-movement) and incorporates the object's lateral motion direction into the horizontal component of the self-movement direction for heading estimation, resulting in a bias in heading estimation, as reported by previous studies (Layton & Fajen, 2016a; Layton & Fajen, 2016b; Li et al., 2018; Royden & Hildreth, 1996; Warren & Saunders, 1995).

The causal inference model has provided insights into how scene-relative object motion judgment affects heading estimation. However, for heading perception in the presence of an independently moving object, there are two distinct scenarios. One is as tested in Dokka et al. (2019), that is, the laterally moving object is placed in the world thus it approaches the observer while moving laterally in the scene. In this case, the heading bias is in the opposite direction of object lateral motion (Koerfer & Lappe, 2020; Layton & Fajen, 2016a; Layton & Fajen, 2016b; Li et al., 2018; Warren & Saunders, 1995). The other is that the object moves laterally in the scene but does not simultaneously approach the observer. This is similar to a common everyday scenario in which a person is walking forward while another person ahead moves at the same speed but also shifts sideways, either to the left or right. Such non-approaching stimuli are classic scenarios for studying heading perception in the presence of moving objects (Royden & Hildreth, 1996; Royden & Hildreth, 1999) or biological motion (Koerfer & Lappe, 2020; Mayer, Riddell, & Lappe, 2021). In this case, the heading bias is in the same direction of the object lateral motion (Koerfer & Lappe, 2020; Li et al., 2018; Royden & Hildreth, 1996; Royden & Hildreth, 1999). Given that Dokka et al. (2019) exclusively focused on the approaching-object scenario, it remains unclear whether the causal inference model can account for data from the non–approaching-object scenario.

### Current study

The current study has three central aims. First, we examined the effects of various visual cues on scene-relative object motion judgment and concurrent heading estimation from optic flow. The goal was to shed light on the relationship between these two processes through the effects of visual cues. Experiment 1 examined the effects of visual cues such as object position offset from the background FoE, self-movement speed, and the binocular disparity. Experiment 2 took a different angle and examined how increasing dot density inside the object window to increase object motion signals affected scene-relative object motion direction judgment and heading estimation. If varying a specific visual cue brings about similar effects on scene-relative object motion judgment and heading estimation, this would indicate that these two processes show similar dependence on visual cues and thus are coupled. In contrast, if varying a specific visual cue brings about different effects on these two processes, this would suggest that they operate independently.

**Figure A1.**
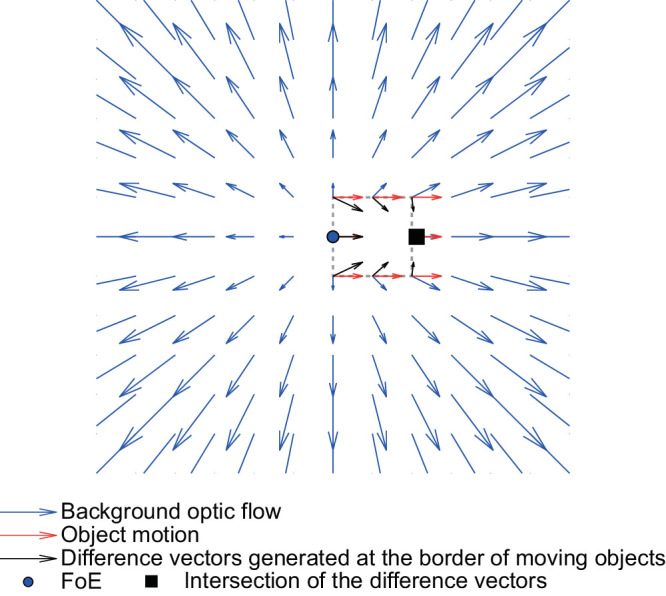
Illustration of the motion difference vectors (black vectors) at the border of a moving object (gray dashed lines) and the motion vectors in the background optic flow (blue vectors). The moving object maintains a constant distance from the observer while moving rightward (red vectors). The difference vectors are generated by subtracting the motion vectors in the background optic flow from the object motion vectors. The difference vectors intersect at a point (black square) to the right of the actual FoE (blue circle) of the background optic flow. Accordingly, for a computational model that pools the difference vectors at the border of the moving object and those in the background flow for heading estimation, the bias in heading estimation is in the same direction of the object lateral motion.

Second, we examined whether the causal inference model can account for data from the non–approaching-object scenario. As in Dokka et al. (2019), we varied the object motion speed and tested participants with a dual-task paradigm, that is, participants were asked to judge the object motion direction in the scene and also their perceived heading at the end of the presented visual motion. Our visual motion display simulated forward self-movement through a three-dimensional (3D) cloud composed of random dots in the presence of an independently moving object also composed of random dots. The object moved laterally in the scene, but also moved with the observer to keep a constant distance from the observer. To explain the heading bias induced by this non-approaching moving object, we incorporated the difference vectors model (Royden, 2002) into the causal inference framework. Specifically, Royden (2002) proposed that heading biases arise when observers pool the motion difference vectors at the borders of the moving object and in the background optic flow for heading estimation (see Figure A1 in the Appendix). The advantage of this model is that it can account for the direction of heading biases caused by both approaching and non-approaching object motion.

**Figure 1.**
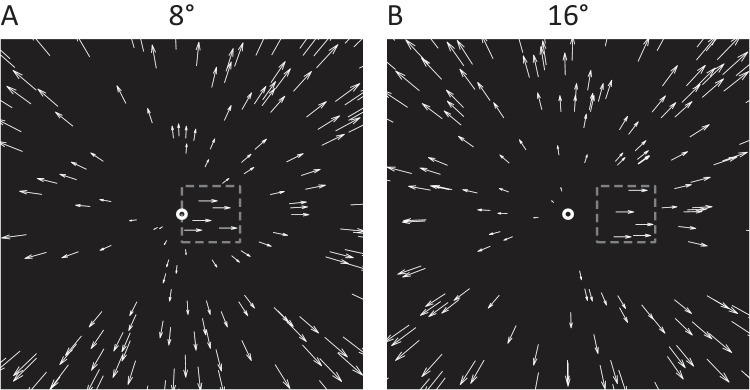
Schematic illustrations of the visual stimuli. (**A**) The 8° condition: the dots inside the opaque window moved rightward, and the opaque window's position offset is at 8° to the right of the background FoE (Supplementary Movie S1A). (**B**) The 16° condition: the dots inside the opaque window moved rightward, and the opaque window's position offset is at 16° to the right of the background FoE (Supplementary Movie S1B). The center white circle (absent in the experiment) indicates the heading direction of the simulated observer translation (i.e., the FoE of the background optic flow). The boundaries of the opaque window (absent in the experiment) are shown for illustration purposes only. The movies are optimized for viewing in Chrome or Firefox browsers. Safari users may need to download the files for proper playback.

Third, we addressed the confounding issue of speed and position of the moving object. In many of the previous studies (Layton & Fajen, 2016a; Layton & Fajen, 2016b; Royden & Hildreth, 1996; Warren & Saunders, 1995), object position and object speed were intertwined, that is, fast-moving and slow-moving objects traveled different distances in the visual field over the same duration. It is thus difficult to distinguish whether heading biases were due to object speed or position change in those studies. To disentangle the effects of object position change and speed cues on object motion judgment and heading estimation in the current study, we adopted the experimental paradigm developed by Li et al. (2018) by fixing the position of an opaque window in the display and varied the motion speed of dots inside the window (Figure 1). This allowed for comparisons of object motion direction judgment and heading estimation with objects of different speeds at the same position and objects of the same speed at different positions, thus separating the effects of position change and speed of the moving object on both processes.

## Experiment 1

In this experiment, we examined how varying three visual cues—object position offset from the FoE, simulated self-movement speed, and the binocular disparity—affected scene-relative object motion direction judgment and heading estimation from optic flow. Our goal was to test whether these two processes are coupled or operate independently.

When an object is positioned farther from the FoE, it carries a stronger self-movement–induced retinal motion component, making it more difficult to distinguish from the background flow. If heading estimation and object motion judgment are coupled, increased object offset should impair performance in both tasks. Similarly, faster self-movement speed produces a stronger self-motion–induced retinal motion component in the object, increasing the difficulty of segregating object motion from the background flow. Under the coupled hypothesis, higher self-movement speeds should thus reduce the accuracy of both object motion judgment and heading estimation. In contrast, the binocular disparity enhances depth perception and has been shown to improve scene-relative object motion judgments (Gogel, 1990; Rushton et al., 2007; Warren & Rushton, 2009). If the two processes are coupled, the addition of the binocular disparity cue should also enhance heading estimation performance.

In addition, we examined whether heading errors vary systematically with object speed and object motion direction judgment, as predicted by causal inference theory. Dokka et al. (2019) previously examined the relationship between object motion judgment and heading estimation by varying the speed of laterally moving objects in the scene and asking participants to report whether the object appeared to be stationary or moving in the scene. The underlying assumption is that, when observers could reliably perceive a moving object, the visual system would likely attribute the image motion to two sources. In contrast, when observers could not perceive a moving object in the scene, the visual system would likely attribute all image motion to a single source. Because presence/absence judgments are susceptible to response bias, that is, observers being more likely to favor one response over the other, the observed relationship between heading estimation and object-motion judgment in their study may reflect not only causal inference, but also such a bias.

To reduce the effect of this response bias and increase sensitivity to the underlying perceptual computations, in the current experiment we modified the object motion judgment task. Rather than asking observers to make a stationary/moving judgment, we asked them to judge the direction of the object's scene-relative motion (leftward or rightward) and measured the percentage of correct responses. This task refinement was motivated by previous findings showing that the perceived direction of object motion in the scene determines the direction of heading biases (Li et al., 2018; Royden & Hildreth, 1996). By also focusing on correct/incorrect judgment, we minimized the effect of response bias and made our design more informative of how heading estimate could be related to the observer's causal inference process.

Within this framework, the key assumption grounded in Bayesian causal inference theory for the current study is that when observers could reliably (i.e., ≥75% accuracy) judge the direction of object motion in the scene, the visual system would likely attribute object and background motion to distinct sources and rely on background optic flow alone for heading estimation, resulting in minimal heading bias. In contrast, when observers could not reliably judge the direction of object motion in the scene, the visual system would likely treat the object motion as part of the global optic flow field, that is, arising from a single source, and thus incorporate it into the heading computation, resulting in a systematic bias in the direction of object motion by performing the computation as shown by the motion difference vectors model (Royden, 2002; see Appendix for the details).

This assumption generates several behavioral predictions. Specifically, if heading biases only occur when object motion is misattributed to self-movement, then heading errors should increase and then decrease with object motion speed, and approach zero at object motion speeds when scene-relative object motion judgments rearch 100% accuracy. In addition, when observers could reliably judge scene-relative object motion, trials with incorrect object direction judgments should show larger heading errors than correct trials. Last, heading errors should be greatest at object speeds where observers could not reliably judge scene-relative object motion. If the behavioral data deviate from these predictions, it would falsify the causal inference assumption.

### Participants

Sixty-seven students aged 18 to 31 years (all naïve as to the purpose of the study except one author) at East China Normal University participated in this experiment. Participants were divided into four groups of 20 participants for each of the four display conditions. The author participated in four groups. One participant participated in groups 1, 2, and 3; one in groups 3 and 4; one in groups 1 and 2; two in groups 2 and 4; and four in groups 1 and 3. As a result, group 1 had 7 male and 13 female participants with an average age of 22.3 ± 2.47 years, group 2 had 8 male and 12 female participants with an average age of 22.70 ± 2.47 years, group 3 had 10 male and 10 female participants with an average age of 21.95 ± 3.14 years, and group 4 had 9 male and 11 female participants with an average age of 22.25 ± 2.88 years. All participants had normal or corrected-to-normal vision and provided written consent before the experiment started. This research was approved by the Institutional Review Board at NYU Shanghai. We determined the sample size based on previous research (e.g., Li et al., 2018).

### Visual stimuli and apparatus

The display simulated an observer translating through a 3D cloud composed of 162 non-expanding white dots (diameter, 0.26°) with the field of view (90° horizontal × 90° vertical). Dots were randomly distributed on the image plane with the density of 0.02 dots/deg². They were then back projected into the 3D cloud by random sampling of the depth in the range of 0.56 to 5.00 m. During the course of a trial, when a dot left the field view, it was regenerated at a random position on the image plane to keep the dot density and the number of visible dots constant throughout the trial. An object consisting of five non-expanding white dots moved leftward or rightward within an opaque window (16° × 16°) that had a fixed position in the image plane. The density of the object dots matched that of the background dots. The background was black (Figure 1).

The dots within the opaque window were positioned on a fronto-parallel plane located at 1.58 m away from the observer. This plane moved in synchrony with the simulated forward translation of the observer, ensuring that the dots remained at a constant distance of 1.58 m from the observer. Dots moved laterally (leftward or rightward) on the plane at one of six possible speeds: 0 m/s, 0.05 m/s, 0.1 m/s, 0.2 m/s, 0.4 m/s, or 0.8 m/s. As a result, the object's simulated motion in the scene was the combination of motion in depth at the same speed of the observer's translation and their lateral motion on the plane. This resembled a common situation in which a person is walking forward while another person ahead moves at the same speed but also shifts sideways. When a dot within the opaque window moved out of the window, it was regenerated at a random position inside the window at the same depth.

We tested five heading directions (0°, ±10°, ±20°) with negative values to the left of the center of the display and positive values to the right. The center of the opaque window was positioned at the horizontal midline of the display, with a constant offset of either 8° (Figure 1A and Supplementary Movie S1A) or 16° (Figure 1B and Supplementary Movie S1B) to the left or right of the heading direction (i.e., the FoE of the background optic flow). At the 8° offset, the edge (invisible) of the opaque window obscured the background FoE, whereas at the 16° offset, the background FoE remained visible throughout the trial.

We tested two simulated observer translation (i.e., simulated self-movement) speeds (0.5 and 1.0 m/s) with 2D and 3D displays. This resulted in four display conditions: (1) 0.5 m/s 2D, (2) 0.5 m/s 3D, (3) 1 m/s 2D, and (4) 1 m/s 3D. The 3D displays were the same as the 2D displays except that observers viewed the displays through a pair of LCD shutter glasses (Nvidia 3D Vision 2) driven by an infrared emitter connected to the computer. The left and right eye images were temporally interleaved and displayed in synchrony with the opening and closing of the left and right eye shutter glass lenses to create a stereoscopic presentation. As a result, although the depth of the moving object was not specified in the 2D displays, it was clearly specified by the binocular disparity cue in the 3D displays. That is, although observers might perceive the moving object as placed in front of the background optic flow in the 2D displays, they would perceive the moving object as placed in the middle of the 3D cloud in the 3D displays.

The displays were programmed in MATLAB using Psychophysics Toolbox 3 with an ASUS workstation and a NVIDIA GeForce GTX970 graphics card. The frame rate was 60 Hz in 2D mode and 120 Hz (60 Hz per eye) in 3D mode. They were rear-projected on a large screen (101° horizontal × 91° vertical) using a BENQ projector (resolution: 1,280 × 720 pixels) with the refresh rate synced to the frame rate. Participants viewed the display binocularly with the head stabilized by a chin rest at a viewing distance of 56.5 cm.

### Procedure

On each trial, the first frame of the display showed a red fixation point (diameter, 0.26°) at the center of the display for 0.5 second. For 3D displays, the duration of the first frame was extended to 1 second to facilitate binocular fusion. The fixation point then disappeared, followed by the simulated observer translation for 1 second. At the end of the displayed motion, participants performed two tasks. First, they were asked to indicate the direction (left or right) of the object's lateral motion in the scene by pressing the corresponding arrow key on the keyboard (the scene-relative object motion judgment task). Then, they were asked to indicate the direction of their perceived heading by using the mouse to move a vertical bar that appeared in a random position within 20° of the display's center along a white horizontal line at the midline of the display (the heading estimation task). This task sequence allowed us to examine how the perceived direction of object lateral motion in the scene affected the direction of heading bias.

**Figure A4.**
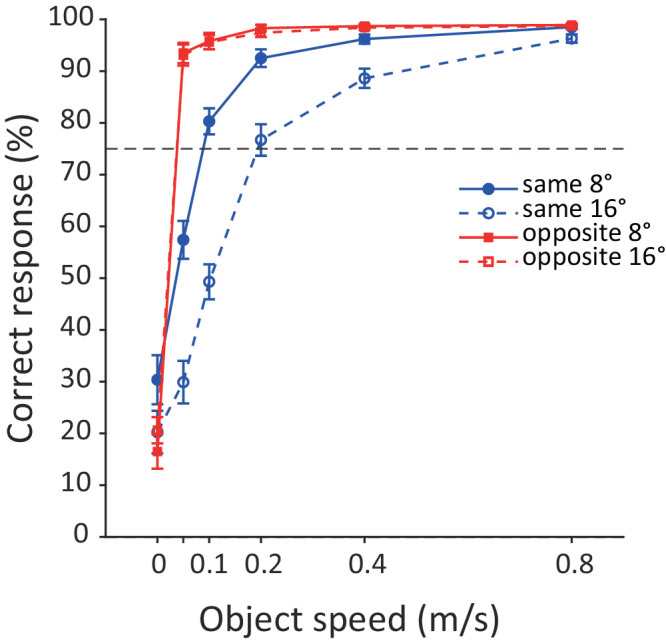
Pilot experiment data. Mean correct response rate (%) of judging scene-relative object motion direction averaged across participants (*N* = 20) as a function of object speed for the four object position offsets. Blue solid and dashed lines represent same 8° and 16° object position offsets, respectively. Red solid and dashed lines represent opposite 8° and 16° object position offsets, respectively. Error bars represent ±1 standard error across participants.

During the experiment, participants were instructed to look at the overall optic flow field rather than any specific local region. Before conducting the experiments, we ran a pilot experiment using the 1 m/s observer translation speed with a 2D display to test all combinations of object positions relative to the FoE (both left and right) and motion directions across all speeds (−0.8 m/s to 0.8 m/s). We found that, for the conditions where the object motion direction matched its position relative to the FoE (e.g., an object located on the right side of FoE moving to the right), the correct response for object motion judgment increased with the object speed much more slowly than for the conditions where the background optic flow moved in the opposite direction to the object motion (e.g., an object located on the right side of the FoE moving to the left). For the latter two conditions, the correct response was suprathreshold (>80%), even at the smallest non-zero object speed of 0.05 m/s, making it difficult to evaluate the causal inference model (see Figure A4 in the Appendix). Accordingly, we tested object position offsets in which the dots in the object window moved laterally in the same direction as the background optic flow (as shown in Figure 1).

A possible concern was that participants might infer the object's lateral motion direction from its screen position rather than from motion cues, but this was unlikely. First, the object position was defined relative to the FoE (could be to the left or right of the center of the screen), rather than simply being in the left or right hemifield of the screen. For example, when the heading was −20°, an object positioned at 8° or 16° to the right of the FoE would appear at −12° or −4°, which was in the left hemifield of the screen. Accordingly, observers could not correctly determine its motion direction solely based on whether it was in the right or left hemifield of the screen. Second, the object was composed of random dots with the same density as those in the background optic flow with no visible boundary. Observers would not be able to see the object before it started moving, making it unlikely for participants to infer the object's motion direction based on its position.

Before the start of the experiment, participants completed practice trials with the optic flow displays without a moving object to become familiar with the heading estimation task. In each set of practice trials, there were 11 heading directions (0°, ±5°, ±10°, ±15°, ±20°, and ±25°), and each heading direction had five trials. All practice trials were randomized. Participants received two to three sets of practice trials until the mean absolute heading error for each heading direction was less than 5°. Participants then completed 40 practice trials randomly selected from the experimental trials before the data collection. No feedback was provided in the practice or the experimental trials to prevent participants from developing artificial strategies or heuristics that could distort their natural perceptual responses.

Each display condition had 120 experimental conditions (4 object position offsets × 6 object speeds × 5 heading directions). Each experimental condition had five trials. Thus, there were a total of 600 randomized trials in each display condition. Based on the data from the pilot experiment and the fact that participants had plenty of practice to ensure they could judge heading from optic flow with the accuracy within 5° before data collection, we determined that five trials per condition were sufficient. The testing of each display condition took approximately 40 minutes to complete, and each participant group viewed only one display condition (i.e., a between-subjects design was used in this experiment).

### Data analysis

For the scene-relative object motion direction judgment, we calculated the percentage of correctly judging the object lateral motion direction in the scene. For the heading estimation, we calculated the heading error, defined as the angle between the perceived and the actual heading directions. To examine how performance on the scene-relative object motion judgment and the heading estimation tasks changed with object speed, we collapsed the percentage of correct responses and the heading error data across the five heading directions. We also collapsed the data across leftward and rightward object motion directions. Before collapsing, for each display condition, we conducted a 2 (direction, left/right) × 6 (object speed) repeated-measures analysis of variance (ANOVA) on both the percentage of correct response and heading error data at the 8° and the 16° position offset, respectively. For the percentage of correct responses, neither the main effect of object motion direction nor the interaction between direction and object speed was statistically significant, *F*s ≤ 2.74, *p*s ≥ 0.051, supporting data combination across left and right directions. For heading errors, the main effect of object motion direction was significant only at the 16° position offset for the 1 m/s 2D and the 0.5 m/s 3D display conditions, *F*_(1,19)_ = 5.36, *p* = 0.032 & *F*_(1,19)_ = 4.72, *p* = 0.043, respectively, whereas the interaction effect of direction and object speed remained non-significant, *F*s ≤ 0.91, *p*s ≥ 0.48. For all the other ANOVAs, neither the main effect of object motion direction nor the interaction effect of direction and object speed was statistically significant, *F*s ≤ 4.36, *p*s ≥ 0.051. These results suggest that the influence of object motion direction (left vs. right) on heading errors was consistent across different object speeds. Because our primary interest was in how heading error varied as a function of object speed, regardless of its motion direction, we collapsed the heading error data across left and right object motion directions in subsequent analyses. This resulted in 12 experimental conditions (2 object position offsets × 6 object speeds) for each display condition.

To quantify participants’ ability to judge scene-relative object motion direction, we fitted a cumulative Gaussian function to the percentage of correct responses at each object speed for each participant at 8° and 16° position offsets, respectively. We used the psignifit toolbox, a MATLAB software package, that implemented Wichmann and Hill's maximum-likelihood method (Wichmann & Hill, 2001), for this purpose. We defined the threshold speed of scene-relative object motion direction judgment as the object speed corresponding with a 75% correct response rate to make sure participants could reliably judge scene-relative object motion direction at this speed. This threshold speed reflects the sensitivity limit for judging scene-relative object motion direction. A lower threshold speed indicates greater sensitivity to object motion direction in the scene, driven primarily by flow parsing but with potential contributions from other visual mechanisms.

To evaluate whether the data were consistent with the predictions of the causal inference model, we first examined the effects of object speed on heading estimation at the two object position offsets. The model predicts that heading errors should initially increase and then decrease with object speed, approaching zero at the point where judgments of object motion direction reach near-perfect accuracy. We conducted a 6 (object speed) × 2 (position offset) repeated-measures ANOVA on heading errors to test the statistical significance of any differences for each display condition. For any significant interaction effects, we conducted Tukey honest significant difference (HSD) tests to identify any significant differences between object speeds at the 8° and 16° position offsets. When a repeated-measures factor had three or more levels, we checked sphericity using Mauchly's test. If sphericity was violated, we reported Greenhouse-Geisser corrected degrees of freedom and *p* values when *ε* was less than 0.75 or Huynh–Feldt corrected values when *ε* was greater than 0.75.

For each participant, we then identified the object speed closest to the threshold speed (i.e., 75% correct response rate) of scene-relative object motion direction judgment. We divided heading errors at this speed into two categories: one corresponding with correct responses and the other with incorrect responses. The causal inference model predicts that heading errors should be smaller for correct than for incorrect responses at this speed. We conducted a 2 (position offset) × 2 (response category) repeated-measures ANOVA on heading errors to examine whether the data supported the causal inference model.

Considering individual differences in causal inference, we also plotted histograms of the object speed corresponding with the largest heading error in each participant's data. If, for most participants, the object speed at which the heading error reached its maximum is equal to or greater than the speed at which participants could reliably judge the object motion direction in the scene, this would provide strong evidence against the causal inference model.

### Results

#### Judgments of scene-relative object motion direction

**Figure 2.**
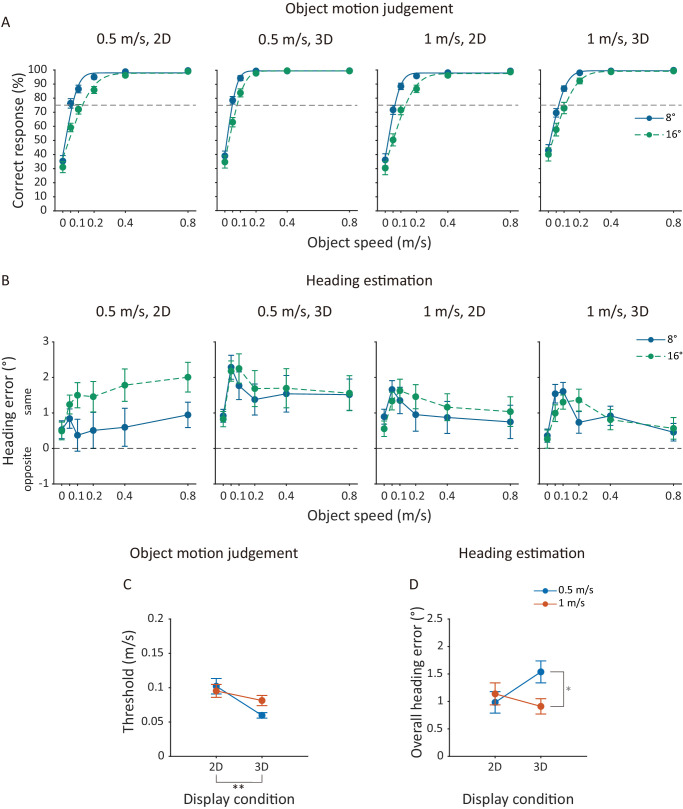
Experiment 1 data. (**A**) Mean percentage of correct responses (%) of judging scene-relative object motion direction and (**B**) mean heading error averaged across participants as a function of object speed at 8° and 16° object position offsets. Each panel plots the data from one of the four display conditions. Solid and dashed lines in (**A**) represent the cumulative Gaussian curves fitted to the mean data. Positive heading errors in (**B**) and (**D**) indicate a heading bias in the same direction as the object's lateral motion in the scene and negative heading errors indicate a bias in the opposite direction. (**C**) Mean threshold speed of scene-relative object motion direction judgment and (**D**) mean overall heading error averaged across participants against the display conditions for the two self-movement speeds. Error bars represent ±1 standard error across 20 participants. *p < 0.1; **p < 0.01.

Figure 2A plots the mean percentage of correct responses for judging scene-relative object motion direction, averaged across 20 participants, against object speed for the 2 object position offsets and the 4 display conditions. Solid and dashed lines represent the cumulative Gaussian curves fitted to the mean data. At zero object speed, participants perceived the object as moving in the opposite direction of the background flow, resulting in a correct response percentage of less than 50%. This bias likely results from a combination of induced motion caused by local motion signals in the background optic flow and more importantly a global subtraction process associated with flow parsing, as described by Warren and Rushton (2009). Although the percentage of correct responses increased with object speed at both 8° and 16° object position offsets, it reached 100% faster at 8° than 16° position offset, indicating a greater sensitivity to the object motion direction in the scene at an object position offset closer to the background FoE.

Given the similar effects of visual cues on the data at the two object position offsets, we collapsed the data across the two position offsets to increase statistical power for the examination of the between-subjects effects of visual cues such as the binocular disparity and self-movement speed on scene-relative object motion direction judgment. Figure 2C plots the mean threshold speed of scene-relative object motion direction judgment, averaged across 20 participants, against display condition for the two self-movement speeds. We conducted a 2 (display condition) × 2 (self-movement speed) independent-measures ANOVA to test the statistical significance of any differences between the four display conditions. Across the two self-movement speeds, the threshold speed was significantly lower for the 3D than the 2D display condition—main effect of display condition, *F*_(1, 156)_ = 11.12, *p* = 0.0011, $\eta_{p}^{2}$ = 0.066—indicating that adding the binocular disparity cue to the display significantly increased the sensitivity to the direction of object motion in the scene.

#### Heading estimation

Figure 2B plots the mean heading error, averaged across 20 participants, against object speed for the two object position offsets and the four display conditions. Consistent with previous findings on heading estimation in the presence of a non-approaching laterally moving object (Li et al., 2018; Royden & Hildreth, 1996), for all four display conditions, the mean heading error was in the same direction as the object's lateral motion at all object speeds and position offsets.

The effects of object speed and object position offset on heading estimation varied with display conditions. Regarding the effect of object speed, for the 0.5 m/s 2D display condition, heading error increased with object speed at the 16° object position offset, but this increasing trend was not significant at the 8° offset—interaction effect of object position offset and object speed, *F*_(5, 95)_ = 4.06, *p* = 0.0022, $\eta_{p}^{2}$ = 0.18. In contrast, for the 1 m/s 3D display condition, at both object position offsets, heading error quickly increased and then decreased with object speed—main effect of object speed, *F*_(2.61, 49.58)_ = 7.50, *p*_(Greenhouse-Geisser)_ < 0.001, $\eta_{p}^{2}$ = 0.28; Tukey's HSD tests: 0 m/s vs. 0.1 m/s, *p* < 0.001, 0.1 m/s vs. 0.8 m/s, *p* = 0.0010. In addition, the larger object position offset (16°) delayed the occurrence of the largest heading error—interaction effect of object position offset and object speed, *F*_(3.28, 62.31)_ = 4.66, *p*_(Greenhouse-Geisser)_ < 0.001, $\eta_{p}^{2}$ = 0.20; Tukey's HSD tests: 0 m/s vs. 0.1 m/s, *p* < 0.001 and 0.1 m/s vs. 0.8 m/s, *p* < 0.001 for the 8° object position offset; 0 m/s vs. 0.2 m/s, *p* < 0.001 and 0.2 m/s vs. 0.8 m/s, *p* = 0.0025 for the 16° object position offset. Specifically, the heading error reached its maximum at 0.1 m/s for the 8° object position offset and at 0.2 m/s for the 16° object position offset. Similar trends (heading error first increased to a peak and then decreased) were observed for the 0.5 m/s 3D and 1 m/s 2D display conditions, but did not attain statistical significance.

Regarding the effect of object position offset, for the 0.5 m/s 2D display condition, across all object speeds, heading error was smaller at the 8° than the 16° object position offset—main effect of position offset, *F*_(1, 19)_ = 14.91, *p* = 0.0011, $\eta_{p}^{2}$ = 0.44. However, no significant difference in heading error was found between the two object position offsets for the other three display conditions.

To examine the effects of visual cues such as the binocular disparity and self-movement speed on the accuracy of heading estimation, we first conducted a 4 (display condition) × 6 (object speed) mixed-design ANOVA on heading errors for 8° and 16° position offsets, respectively. The interaction effect between object speed and display condition was not statistically significant, *F*s_(15, 380)_ ≤ 1.34, *p*s ≥ 0.17. Accordingly, we collapsed heading error data across object speeds for further analysis on the effect of visual cues on heading judgments. We then conducted a 2 (display condition) × 2 (self-movement speed) independent-measures ANOVA on the collapsed overall heading error to test statistical significance of any differences between the four display conditions. Figure 2D plots the mean overall heading error, averaged across participants, against display conditions for the two self-movement speeds. Given the significant interaction effect of display condition and self-movement speed, *F*_(1, 156)_ = 4.45, *p* = 0.036, $\eta_{p}^{2}$ = 0.028, we conducted Tukey HSD tests and found that, although the heading error was similar for the two self-movement speeds in the 2D display condition (*p* = 0.94), the heading error showed a trend to be greater at the self-movement speed of 0.5 m/s than 1 m/s in the 3D display condition (*p* = 0.078), indicating that the effect of adding the binocular disparity cue to the display on heading estimation depends on self-movement speed.

#### Causal inference analysis

**Figure 3.**
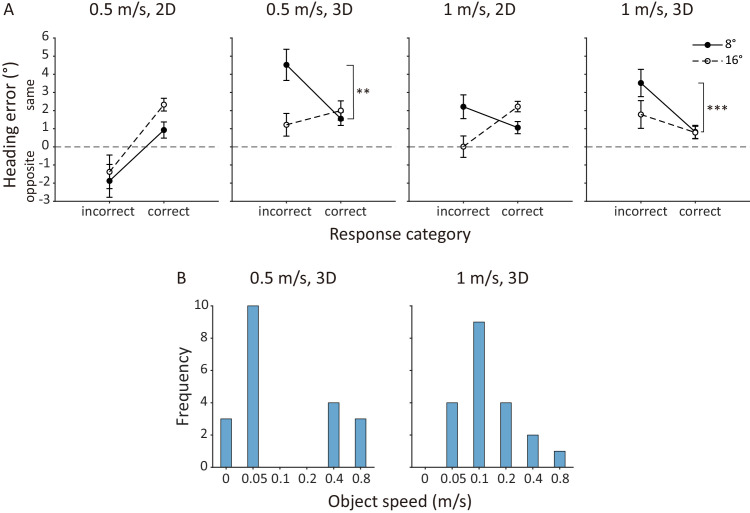
Experiment 1 causal inference analysis results. (**A**) Mean heading error averaged across participants against the two response categories. Each panel plots the data from each of the four display conditions. Error bars represent ±1 standard error across 20 participants. **p < 0.01, ***p < 0.001. (**B**) Frequency of object speed at which heading error peaked in each participant's data at the 8° object position offset for the 0.5 m/s 3D (left) and the 1 m/s 3D (right) display conditions.

Figure 3A plots the mean heading error, averaged across participants, against response category. For the 0.5 m/s 2D display condition, at both the 8° and 16° object position offsets, the heading error was in opposite directions for incorrect versus correct responses—main effect of response category, *F*_(1, 19)_ = 11.95, *p* = 0.0026, $\eta_{p}^{2}$ = 0.39. For the other three display conditions, given the significant interaction effect of object position offset and response category, *Fs*_(1, 19)_ ≥ 4.64, *p* ≤ 0.044, $\eta_{p}^{2}$ ≥ 0.20, we carried out Tukey HSD tests and found that only for the 3D display condition at the 8° object position offset was the heading error for incorrect responses higher than for correct responses (*p* = 0.0058 and *p* = 0.00076 for the 0.5 m/s 3D and 1 m/s 3D display conditions, respectively), consistent with the predictions of the causal inference model.

Figure 3B plots the frequency of object speed at which heading error reached its maximum in each participant's data for these two display conditions at the 8° object position offset. For the 0.5 m/s 3D display condition, the most frequent object speed with the peak heading error (0.05 m/s) was closely aligned with the mean threshold speed for scene-relative object motion direction judgment (mean±*SE*: 0.045 ± 0.018 m/s). For the 1 m/s 3D display condition, the most frequent object speed with the peak heading error increased to 0.1 m/s, which was above the mean threshold speed for scene-relative object motion direction judgment (0.061 ± 0.025 m/s). This finding indicates that, even for these two display conditions where we observed smaller heading errors for correct than incorrect responses for scene-relative object motion direction judgments near the threshold speed, the greatest heading errors most frequently occurred when participants could reliably judge the direction of object motion direction in the scene. This finding is inconsistent with the predictions of the causal inference model.

### Discussion

For the scene-relative object motion direction judgment task, the percentage of correct responses increases with object speed and reaches 100% faster at the 8° than the 16° object position offset, indicating a reduced sensitivity to scene-relative object motion direction at the larger object position offset. This finding is consistent with our predictions that objects farther away from the FoE in the background flow have higher self-movement components that need to be parsed out, thus requiring stronger object motion signals (such as faster object speeds) for the visual system to detect its scene-relative motion.

**Figure A5.**
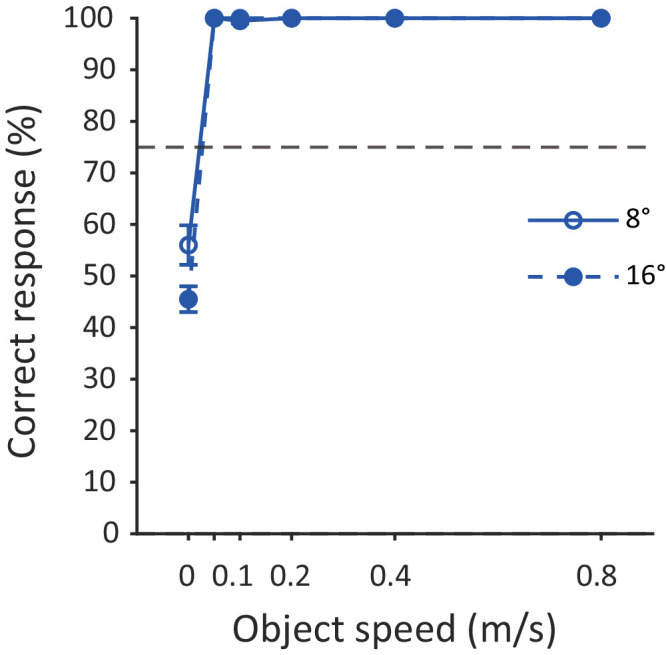
Control experiment data. Mean correct response rate (%) of judging scene-relative object motion direction averaged across four participants as a function of object speed for the 8° and 16° object position offsets. Error bars represent ±1 standard error across participants.

It could be argued that the differences in scene-relative object motion direction judgment between the 8° and 16° object position offsets might be due to eccentricity rather than flow parsing. To address this issue, we conducted a control experiment using the same display setting of the 1 m/s 2D display condition, except that the background dots were stationary. Participants were asked to judge the object motion direction. Except for zero object speed where the correct response rate was about 50-50, participants correctly identified the object motion direction 100% of the time for all the other object speeds at both the 8° and 16° object position offsets (see Figure A5 in the Appendix). This finding indicates that the differences in judging object motion direction in the scene for these two position offsets observed in the current experiment are unlikely due to simple differences in visual localization at different eccentricities and more likely to be due to eccentricity-dependent flow parsing effects.

For the heading estimation task, the effects of both object speed and object position offset were inconsistent across display conditions. Regarding the effect of object speed, although heading error increased with object speed for the 0.5 m/s 2D display condition at the 16° object position offset, it increased and then decreased with object speed for the 1 m/s 3D display condition at both object position offsets. Nevertheless, for the other display conditions, no significant effect of object speed on heading error was found. Regarding the effect of object position offset, although heading error is greater at the 16° than the 8° offset for the 0.5 m/s 2D display condition, there is no statistical difference between the two position offsets in the other three display conditions. Overall, the heading error data are consistent with previous findings that, even without obscuring the FoE, an independent moving object can still bias heading estimation (Li et al., 2018; Riddell, Li, & Lappe, 2019).

Previous studies have reported that adding the binocular disparity cue to the display helps the accurate perception of the object depth, which can lead to increased sensitivity to detect scene-relative object motion during self-movement (Gogel, 1990; Layton & Niehorster, 2019; Rushton et al., 2007; Warren & Rushton, 2009). In this experiment, we observed such an effect across the two tested self-movement speeds.

Surprisingly, the effect of the binocular disparity cue on heading estimation depends on self-movement speed. Without the binocular disparity, heading errors are similar at the two self-movement speeds. With the binocular disparity, heading errors appear to be greater at 0.5 m/s than 1 m/s. The inconsistent effect of the binocular disparity cue on heading estimation has been reported previously. Some studies found that the binocular disparity reduced heading errors (Grigo & Lappe, 1998; Van den Berg & Brenner, 1994), whereas others found no such effect (Li et al., 2018). In the current experiment, the enhancement of scene-relative object motion direction judgment with the binocular disparity at the self-movement speed of 0.5 m/s might have amplified the influence of object motion signals on heading estimation, resulting in larger errors in heading estimation at 0.5 m/s than 1 m/s for the 3D display condition.

In sum, the results of the current experiment show that visual cues such as object speed and object position offset have different effects on scene-relative object motion direction judgment and heading estimation. More important, the binocular disparity cue has contrasting effects on the performance of scene-relative object motion direction judgment and heading estimation. These findings support the claim that scene-relative object motion identification and heading estimation operate as separate processes (Rushton et al., 2018; Rushton, Niehorster, Warren, & Li, 2018; Warren et al., 2012).

According to the causal inference model, heading errors should increase and then decrease with object motion speed, and approach zero at object motion speeds when scene-relative object motion judgments reach 100% accuracy (see Appendix for model simulations). We observed a significant increase and decrease of heading errors with object speed for the 1 m/s 3D display condition. Given that the lack of significance of this trend in other display conditions could be due to the lack of statistical power, we conducted an additional analysis. That is, we examined whether heading errors for incorrect responses were greater than those for correct responses at the threshold speed for scene-relative object motion direction judgment. We observed this trend only for the 0.5 m/s 3D and the 1 m/s 3D display conditions at the 8° object position offset. However, further analysis of individual data showed that, even for these two display conditions, for most participants, heading error reached its maximum at the object speed when participants could reliably judge the direction of object motion in the scene. This finding is inconsistent with the predictions of the causal inference model and indicates that, even when participants could reliably judge scene-relative object motion, object motion signals still affected heading estimation and led to a small but systematic bias in heading judgments.

## Experiment 2

In this experiment, we examined how varying object motion signals through changing the dot density inside the object window affected scene-relative object motion direction judgment and heading estimation. Increasing object motion signals through increasing the dot density inside the object window increases the sensory reliability of object motion signals, which could help the judgment of object motion in the scene. If heading estimation relies on accurate object motion judgment, increased dot density should improve performance in heading estimation.

As in Experiment 1, we examined whether the effect of object speed on heading estimation aligns with the predictions of the causal inference model. Specifically, if heading biases arise when object motion is misattributed to self-movement, heading errors should increase and then decrease with object motion speed, approaching zero at speeds where scene-relative object motion judgments reach near-perfect accuracy. Moreover, when observers can reliably judge scene-relative object motion, trials with incorrect object motion direction judgments should yield larger heading errors than correct trials. Finally, heading errors should be greatest at object speeds where observers could not reliably judge scene-relative object motion.

### Participants

Twenty students and staff (8 males, 12 females; all naïve as to the purpose of the study except one author) aged between 19 and 31 years (mean±*SE*: 22.90 ± 3.02) at East China Normal University participated in this experiment. Among them, eight participants (three males, five females) participated in Experiment 1. All had normal or corrected-to-normal vision and provided written consent before the experiment started. This research was approved by the Institutional Review Board at NYU Shanghai. We determined the sample size based on previous research (e.g., Li et al., 2018).

### Visual stimuli and procedure

The display in this experiment was identical to the 1 m/s 2D display condition in Experiment 1, except that the dot density within the opaque window was varied at three levels, that is, the ratio of dot density in the object window to that of the background optic flow was at 1 (*DR*-1, where *DR* stands for density ratio), 2 (*DR*-2), or 4 (*DR*-4). The experiment setup and the testing procedure were the same as in Experiment 1, except that this experiment tested 360 experimental conditions (4 object position offsets × 6 object speeds × 5 heading directions × 3 object densities). Each condition also had 5 trials, resulting in a total of 1,800 randomized trials. The randomized trials were broken into three sessions with each session lasting approximately 40 minutes.

As in Experiment 1, participants completed 40 practice trials randomly selected from the experimental trials before the testing sessions started. No feedback was provided in the practice or the experimental trials to prevent participants from developing artificial strategies or heuristics that could distort their natural perceptual responses. Participants were encouraged to take breaks in-between the three testing sessions. The entire experiment took approximately 2 hours in total to complete.

### Data analysis

As in Experiment 1, we measured the threshold speed of scene-relative object motion direction judgment and the accuracy of heading estimation. Given the within-subject design of all experimental conditions in this experiment, to examine the effects of object position offset and object density on scene-relative object motion direction judgment, we conducted a 2 (object position offset) × 3 (object density) repeated-measures ANOVA on the threshold to test for statistical significance of any differences.

To evaluate whether the data were consistent with the predictions of the causal inference model, as in Experiment 1, we first examined the effects of object speed on heading estimation at the two object position offsets. We conducted a 6 (object speed) × 2 (position offset) repeated-measures ANOVA on heading errors to test the statistical significance of any differences for each object density level. For any significant interaction effects, we conducted Tukey HSD tests to identify specific differences between conditions. When a repeated-measures factor had three or more levels, we checked sphericity using Mauchly's test. If sphericity was violated, we reported Greenhouse–Geisser corrected degrees of freedom and *p* values when *ε* was less than 0.75, or Huynh–Feldt corrected values when *ε* was greater than 0.75.

We then categorized heading errors corresponding with correct and incorrect responses at the object speed closest to the threshold speed of scene-relative object motion direction judgment. We conducted a 2 (response category) × 3 (object density) repeated-measures ANOVA on heading errors to examine at which object density the data are consistent with the predictions of the causal inference model. As in Experiment 1, we also plotted histograms of the object speed corresponding to the largest heading error in each participant's data to examine whether participants could reliably judge the direction of object motion in the scene at this speed.

### Results

#### Judgments of scene-relative object motion

**Figure 4.**
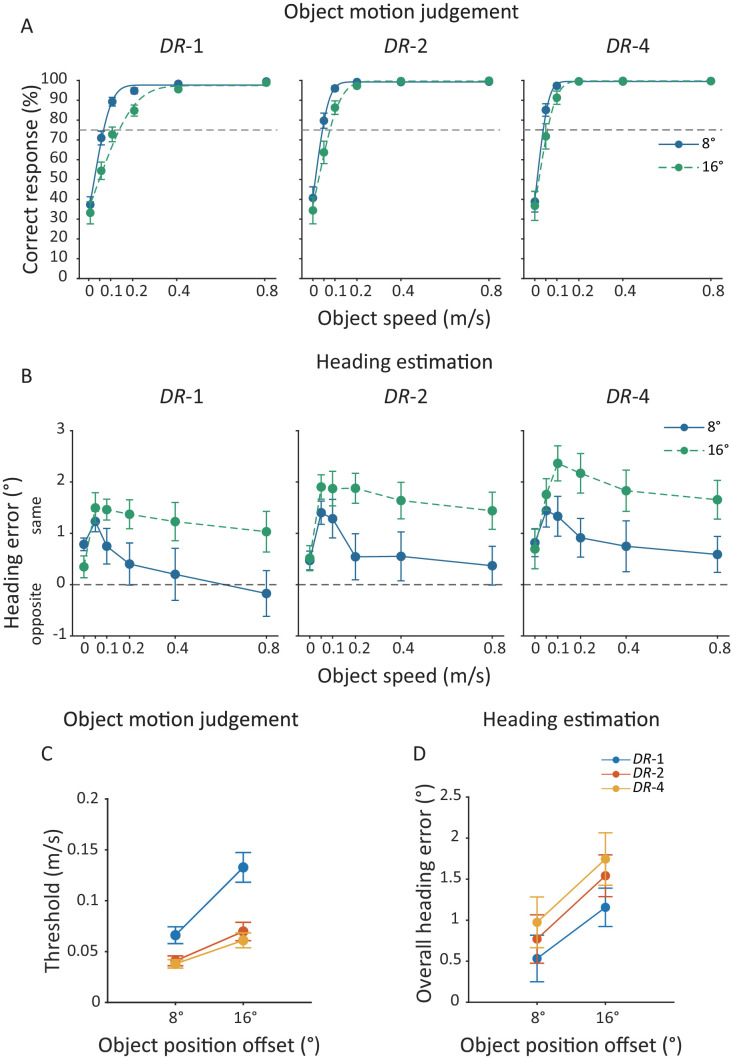
Experiment 2 data. (**A**) Mean percentage (%) of correct responses of judging scene-relative object motion direction and (**B**) mean heading error averaged across participants as a function of object speed at the 8° and 16° object position offsets. Each panel plots the data for one of the three object density levels. Solid and dashed lines in (**A**) represent the cumulative Gaussian curves fitted to the mean data. Positive heading errors indicate a heading bias in the same direction as the object's lateral motion, and negative heading errors indicate a bias in the opposite direction. (**C**) Mean threshold speed of scene-relative object motion direction judgment and (**D**) mean overall heading error averaged across participants against the object position offsets for the three object density levels. Error bars represent ±1 standard error across 20 participants.

Figure 4A plots the mean percentage of correct responses for judging scene-relative object motion direction, averaged across 20 participants, against object speed at the two object position offsets and the three object density levels. Consistent with the results of Experiment 1, the percentage of correct responses increased with object speed and reached 100% faster at the 8° than the 16° position offset, indicating a greater sensitivity to scene-relative object motion direction at the object position offset closer to the background FoE. As we expected, the percentage of correct responses also reached 100% faster with the increase in object density, indicating an increased sensitivity to scene-relative object motion direction with the increase of object motion signals.

Figure 4C plots the mean threshold speed of scene-relative object motion direction judgment, averaged across 20 participants, against object position offset for the three object density levels. Across the two object position offsets, increasing object density significantly decreased the threshold speed—main effect of object density: *F*_(1.03, 14.48)_ = 14.68, *p*_(Greenhouse-Geisser)_ = 0.0016, $\eta_{p}^{2}$ = 0.51. Across the three levels of object density, the threshold speed was higher at the 16° than at the 8° object position offset—main effect of object position offset: *F*_(1, 19)_ = 15.40, *p* < 0.001, $\eta_{p}^{2}$ = 0.45. In addition, object position offset had a greater effect on the threshold speed at the lowest level of object density than the other two levels—interaction effect of object position offset and object density: *F*_(1.32, 18.49)_ = 20.43, *p*_(Greenhouse-Geisser)_ < 0.001, $\eta_{p}^{2}$ = 0.59.

#### Heading estimation

Figure 4B plots the mean heading error, averaged across 20 participants, against object speed at the two object position offsets and the three object density levels. Consistent with previous findings (Li et al., 2018; Royden & Hildreth, 1996) and the results of Experiment 1, the mean heading error was in the same direction as the object's lateral motion at all but one object speed at the 8° position offset.

The effects of object speed and object position offset on heading errors were consistent across the three levels of object density. Regarding the effect of object speed, at all three object density levels, although heading error had a quick jump at the first non-zero object speed and then decreased with object speed at the 8° object position offset, heading error quickly increased, but then did not decrease significantly with object speed at the 16° object position offset—interaction effect of object speed and object position offset: *F*_(3.69, 70.10)_ = 6.13, *p*_(Greenhouse-Geisser)_ < 0.001, $\eta_{p}^{2}$ = 0.24 for DR-1; *F*_(3.76, 71.50)_ = 5.14, *p*_(Greenhouse-Geisser)_ = 0.0013; $\eta_{p}^{2}$ = 0.21 for *DR*-2, and *F*_(3.14, 59.57)_ = 6.59, *p*_(Greenhouse-Geisser)_ < 0.001, $\eta_{p}^{2}$ = 0.26 for *DR*-4; Tukey's HSD tests: 0.05 m/s vs. 0.8 m/s, *p*s ≤ 0.0066 at the 8° object position offset and *p*s ≥ 0.55 at the 16° object position offset. Regarding the effect of object position offset, at all three object density levels, heading error was significantly higher at the 16° than the 8° object position offset across all object speeds—main effect of object position offset: *F*s_(1, 19)_ ≥ 17.74, *p*s < 0.001, $\eta_{p}^{2}$ ≥ 0.48.

To examine the effect of the object density level on heading estimation, we averaged heading errors across object speeds to obtain the overall heading error and then conducted a 2 (object position offset) × 3 (object density) repeated-measures ANOVA on the overall heading error. Figure 4D plots the mean overall heading error, averaged across 20 participants, against the object position offset for the three object density levels. Across the two object position offsets, increasing object density significantly increased heading error—main effect of object density: *F*_(1.13, 21.55)_ = 7.78, *p*_(Greenhouse-Geisser)_ = 0.0088, $\eta_{p}^{2}$ = 0.29. Across the three levels of object density, the heading error was larger at the 16° than at the 8° object position offset—main effect of object position offset: *F*_(1, 19)_ = 25.06, *p* < 0.001, $\eta_{p}^{2}$ = 0.57.

#### Causal inference analysis

**Figure 5.**
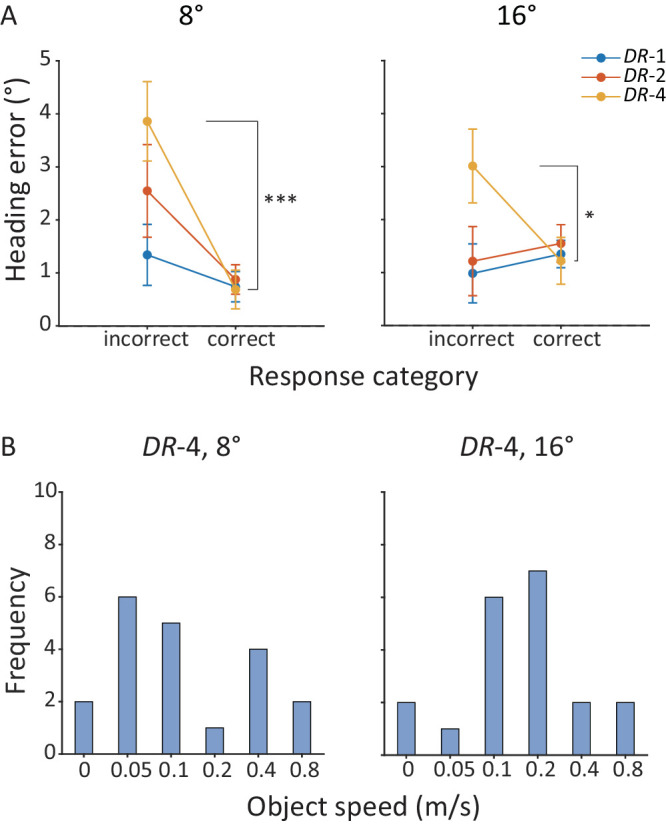
Experiment 2 causal inference analysis results. (**A**) Mean heading error averaged across participants against the two response categories at the three object density levels and the 8° (left) and the 16° (right) object position offsets. Error bars represent ±1 standard error across 20 participants. *p < 0.05, ***p < 0.001. (**B**) Frequency of object speed at which heading error peaked in each participant's data at the highest object density level (*DR*-4) and the 8° (left) and the 16° (right) object position offsets.

Figure 5A plots the mean heading error, averaged across 20 participants, against response category for the three object density levels at the two object position offsets. Given the significant interaction effects between response category and object density at both the 8° and 16° object position offsets, *F*s_(2, 38)_ ≥ 4.02, *p*s ≤ 0.026, $\eta_{p}^{2}$ ≥ 0.17, we carried out Tukey HSD tests and found that only at the highest level of object density (*DR*-4) were heading errors for incorrect responses significantly greater than those for correct responses (*p* = 0.00035 and *p* = 0.040 for the 8° and 16° object position offsets, respectively), consistent with the predictions of the causal inference model.

Figure 5B plots the frequency of object speed with the largest heading error in each participant's data for *DR*-4. The most common object speeds with the peak heading error were 0.05 m/s and 0.1 m/s at the 8° position offset (Figure 5B, left) and 0.1 m/s and 0.2 m/s at the 16° position offset (Figure 5B, right), indicating that the largest heading error happened at higher object speeds at larger object position offsets. Nevertheless, these speeds are notably higher than the threshold speeds of scene-relative object motion direction judgment at the two object position offsets (mean±*SE*: 0.038 ± 0.019 m/s and 0.061 ± 0.033 m/s for the 8° and 16° object position offsets, respectively). This finding indicates that, even at the highest object density level (*DR*-4) where we observed smaller heading errors for correct than incorrect responses in scene-relative object motion direction judgments near the threshold speed, peak heading errors most frequently occurred when participants could reliably judge the object motion direction in the scene. This finding is inconsistent with the predictions of the causal inference model.

### Discussion

Consistent with the results of Experiment 1, for the scene-relative object motion direction judgment task, across the three object density levels, the percentage of correct responses for judging scene-relative object motion direction increases with object speed and reaches 100% faster for the 8° than for the 16° object position offset, as revealed by the larger threshold speed at the 16° than at the 8° object position offset.

For the heading estimation task, at all three object density levels, the heading error increases quickly and then decreases slowly with object speed at the 8° object position offset, but does not appear to decrease with object speed at the 16° object position offset. Across object speeds, the heading error is larger at the 16° than at the 8° object position offset. This result again is consistent with previous findings that an independent moving object can bias heading estimation even without obscuring the FoE (Li et al., 2018; Riddell et al., 2019).

More important, as we predicted, the threshold speed of the scene-relative object motion task decreased with increasing object density level owing to the fact that the increased object motion signals made the direction of object motion in the scene easier to judge. Surprisingly, for the heading estimation task, heading errors increased with object density level, indicating a decrease in the accuracy of heading estimation with the increase in object motion signals. These results show that the effect of increasing object motion signals through increasing object density level on scene-relative object motion direction judgment and heading estimation parallels that of the binocular disparity cue as observed in Experiment 1. Specifically, although increasing object motion signals improved judgments of scene-relative object motion direction, it also increased the bias in heading estimation owing to independent object motion. This finding provides further evidence that scene-relative object motion judgment and heading estimation operate as separate processes (Rushton et al., 2018; Rushton et al., 2018).

Further trial-by-trial analysis showed that only at the highest object density level (*DR*-4) were heading errors smaller for correct than for incorrect scene-relative object motion judgments near the threshold speed. However, even for *DR*-4, heading errors reached their maximum when participants could already reliably judge the object motion direction in the scene. This finding replicates what we observed in Experiment 1 and is consistent with prior findings that heading judgments still exhibit small but systematic biases even when scene-relative object motion is clearly identifiable through cues such as the binocular disparity, color, or biological motion (Li et al., 2018; Riddell et al., 2019; Riddell & Lappe, 2017). Moreover the fact that heading errors peaked at higher object speeds for the 16° than for the 8° object position offset is likely owing to the increased speed of the background optic flow at larger eccentricities, which reduces the relative weight of object motion signals and requires greater object speeds to bias heading estimation. Taken together, these results challenge the generality of the causal inference prediction that accurate object motion judgments should eliminate heading biases.

## General discussion

In this study, by adopting a dual-task paradigm in which participants judged the direction of object motion in the scene and concurrently estimated their perceived heading, we sought to investigate three core questions. First, we examined how different visual cues affect scene-relative object motion judgment and heading estimation, with the goal of understanding the relationship between these two perceptual processes. Second, we tested whether the causal inference framework can account for biases in heading estimation in the presence of non-approaching object motion. Third, we disentangled the contributions of object position and speed, which have been conflated in many prior studies, to clarify their respective roles in driving heading biases.

### Differential effects of visual cues on object motion judgment and heading estimation

Results from both experiments converge on a key finding: visual cues can exert different, and sometimes opposing, effects on scene-relative object motion judgment and heading estimation. For example, although object position offset consistently increased threshold speed in object motion judgment across display conditions, its effects on heading estimation varied with the display conditions. More important, in Experiment 1, although adding visual cues such as binocular disparity to the display improved the sensitivity to scene-relative object motion direction at the self-movement speed of 0.5 m/s, it appeared to have a negative effect on heading estimation. In Experiment 2, we observed similar contrasting effects on scene-relative object motion direction judgment and heading estimation with the object density cue. Specifically, although increasing object motion signals through increasing object density level improved the sensitivity to the object motion direction in the scene, it decreased the accuracy of heading estimation.

These findings suggest that the two processes do not share a common dependence on visual cues. Instead, each process selectively exploits distinct types of information, indicating that scene-relative object motion judgment and heading estimation are dissociable rather than coupled. This conclusion is consistent with earlier work showing that adding laminar flow to a radial flow display changed heading estimation, but had no effect on scene-relative object motion judgment (Warren et al., 2012). Similarly, Rushton et al. (2018) reported that, although form and position cues in optic flow influenced heading estimation, only motion cues contributed to the judgment of scene-relative object motion. Additional work by Rushton et al. (2018) showed that depth range and gaze rotation affected heading estimation, but not scene-relative object motion judgment through flow parsing. By jointly measuring both processes in the same observers, the current study extends this literature and provides direct evidence that scene-relative object motion judgment and heading estimation operate separately, with each process selectively drawing on different types of visual information.

### Neural mechanisms underlying dissociation

Research findings from previous neurophysiological studies provide supporting evidence that scene-relative object motion judgment and heading estimation engage separate neural mechanisms, each selectively processing different types of visual information. In the current study, the selective enhancement of scene-relative object motion judgment by the binocular disparity cue suggests that the brain regions involved in scene-relative object motion judgment respond to both the binocular disparity and object motion during self-movement. Indeed, several human functional magnetic resonance imaging studies have shown that the ventral intraparietal area (VIP) is crucial for detecting scene-relative object motion during self-movement (Calabro & Vaina, 2012; Field, Biagi, & Inman, 2020; Pitzalis et al., 2020; Sulpizio et al., 2024). Additionally, neurophysiological studies have revealed that macaque VIP neurons are tuned to the binocular disparity (Bremmer, Schlack, Kaminiarz, & Hoffmann, 2013; Yang, Liu, Chowdhury, DeAngelis, & Angelaki, 2011).

Although macaque VIP neurons also respond to visual and vestibular signals related to self-movement (Chen, DeAngelis, & Angelaki, 2013; Zhang & Britten, 2004), deactivation of VIP does not disrupt heading estimation (Chen, Gu, Liu, DeAngelis, & Angelaki, 2016), indicating that the VIP is not directly involved in heading estimation. Instead, the VIP is likely involved in using different sources of self-movement information to separate self-movement from object motion components in the optic flow field (Field et al., 2020). The binocular disparity tuning in the VIP facilitates this dissociation, supporting the VIP's primary role in scene-relative object motion judgment rather than heading estimation (Yang et al., 2011).

Further evidence for separate neural mechanisms for scene-relative object motion judgment and heading estimation comes from the distinct functions of the macaque medial superior temporal lateral (MSTl) and dorsal (MSTd) areas in processing object motion and self-movement information. Neurons in the MSTd area have larger receptive fields than neurons in MT and receive inputs from MT neurons that are known to respond to motion signals (Duffy & Wurtz, 1991; Tanaka et al., 1986). Their activity has been shown to be causally linked to the performance of heading estimation from optic flow (Britten & van Wezel, 2002; Gu, DeAngelis, & Angelaki, 2012). In contrast, neurons in the MSTl area have small receptive fields with strong surround suppression and are highly responsive to object motion, but show limited response to large-scale optic flow (Eifuku & Wurtz, 1998; Tanaka, Sugita, Moriya, & Saito, 1993). They are thus likely involved in scene-relative object motion judgment, but not heading estimation. This differentiation in function of the MST neurons can explain why the object density cue had contrasting effects on scene-relative object motion judgment and heading estimation as observed in the current study.

Together, these findings highlight the roles of the VIP, MSTl, and MSTd areas in supporting separate neural mechanisms for scene-relative object motion judgment and heading estimation.

### Causal inference and heading biases with independent object motion

A second goal of the study was to test whether the causal inference framework could account for heading biases induced by non-approaching object motion. Across both experiments, the results indicate that heading errors mostly did not follow the increase–decrease pattern predicted by the model. Instead, object motion signals biased heading estimation even when scene-relative object motion was reliably identified. Moreover, in the few conditions where greater heading errors occurred for incorrect than for correct responses near threshold speed, as the model predicts, further analyses on individual data showed that heading errors consistently reached their maximum at object speeds where participants could reliably judge object motion direction. This pattern echoes earlier findings that salient cues such as the binocular disparity, color, or biological motion, although effectively signaling object motion, do not reduce heading biases (Li et al., 2018; Riddell et al., 2019; Riddell & Lappe, 2017). Together, these results call into question the generality of the causal inference framework in explaining heading biases caused by independently moving objects.

Research on the integration of visual and vestibular cues for the perception of self-movement also reveals inconsistent evidence for the causal inference model that predicts the integration of these cues only when the brain could not tell them apart. For example, some studies found that visual and vestibular signals were integrated despite offsets as large as 90° (De Winkel, Katliar, & Bülthoff, 2015; Rodriguez & Crane, 2019; Rodriguez & Crane, 2020) when the brain could clearly separate them, whereas others reported reduced perceptual biases with increasing visual-vestibular discrepancies (Acerbi, Dokka, Angelaki, & Ma, 2018; De Winkel, Katliar, & Bülthoff, 2017).

It has been proposed that these inconsistencies likely reflect the implausibility of the stimuli used in some experiments, for example, large visual-vestibular discrepancies may suggest unnatural scenarios that are rarely encountered in natural settings (French & DeAngelis, 2020). When stimuli are interpretable as realistic, the causal inference model performs well; otherwise, observers struggle to attribute causes correctly. In our study, object motion was confined within an opaque window to disentangle position change and speed cues that were confounded in earlier work (Dokka et al., 2019; Layton & Fajen, 2016a; Layton & Fajen, 2016b; Royden & Hildreth, 1996; Warren & Saunders, 1995). Thus, our stimuli do not closely mirror everyday conditions, which may explain the lack of support for the causal inference model.

All these results call for different explanations for the small but systematic biases in heading estimation in the presence of independent object motion. The population heading map model (Lappe, 1996; Lappe & Rauschecker, 1995b) may explain our results and also the finding from previous studies showing a small but systematic bias in heading estimation even when object motion in the scene could be clearly identified with salient visual cues (Li et al., 2018; Riddell et al., 2019; Riddell & Lappe, 2017). This model uses a least-squares optimization algorithm (Heeger & Jepson, 1990; Heeger & Jepson, 1992) to integrate velocity signals across the global optic flow field and calculates a heading direction without segmenting moving objects. It aligns with the properties of neurons in the MT and MST areas (Lappe, 1996) and accurately predicts biases in heading estimation, even when the moving object is far from the FoE in the background optic flow (Li et al., 2018).

### Disentangling the contributions of object position and speed

The third aim of this study was to disentangle the effects of object position and object speed, which have often been conflated in prior research. In many earlier paradigms (Dokka et al., 2019; Layton & Fajen, 2016a; Layton & Fajen, 2016b; Royden & Hildreth, 1996; Warren & Saunders, 1995), faster-moving objects also traveled greater distances across the visual field, making it unclear whether heading biases were driven by motion speed or by positional displacement. By adopting the Li et al. (2018) paradigm, in which object motion was confined to a fixed window while dot speed was varied, we were able to examine the independent and combined contributions of speed and position.

Even when position was fixed, changes in object speed still influenced heading estimation, and the strength of this effect can be modulated by the object positional offset. In addition, heading errors were at least as large at the 16° offset as at the 8° offset, even when the object did not obscure the FoE in the background flow. This finding replicates and extends earlier work showing that independently moving objects bias heading perception through global pooling mechanisms (Li et al., 2018; Riddell et al., 2019). Together, these results clarify a long-standing ambiguity in the literature: heading biases in the presence of independently moving objects cannot be attributed solely to object speed or to position, but arise from their interaction with the background optic flow.

### Broader implications and future directions

Taken together, our results underscore that scene-relative object motion judgment and heading estimation operate separately, with differential reliance on visual cues and dissociable neural substrates. They also highlight the limitations of causal inference as a general framework for explaining heading perception in the presence of object motion. The failure of salient segmentation cues examined in the current study (such as the binocular disparity and object density) as well as in previous studies (such as color and biological motion cues; Li et al., 2018; Riddell et al., 2019; Riddell & Lappe, 2017) to reduce heading biases suggests that heading estimation relies on global pooling strategies rather than the causal segregation of object and self-motion signals.

Note that the non-approaching object motion tested in our study and the approaching object motion used in Dokka et al. (2019) both varied only the lateral speed of object motion in the scene. In Dokka et al.’s study in particular, when the lateral speed was set to zero, the object remained stationary relative to the scene. In contrast, object motion in real-world settings often contains both lateral and approaching components. For example, avoiding oncoming pedestrians or reacting to approaching vehicles typically involves motion that combines a lateral speed with an approaching speed that is independent of self-movement. As such, future research could extend the causal inference framework by jointly manipulating both lateral and approaching speeds, thereby testing the model in more ecologically valid, everyday scenarios.

## Conclusions

This study examined how various visual cues influence scene-relative object motion judgment and concurrent heading estimation. The goal was to reveal the relationship between these two processes. The results showed that object speed and object position offset consistently affected scene-relative object motion judgment, but had inconsistent effects on heading estimation, which were modulated by specific visual cues.

Notably, visual cues such as the binocular disparity and object density level enhanced scene-relative object motion direction judgment but reduced the accuracy of heading estimation. Interestingly, in most cases, heading error reached its maximum at object speeds where observers could reliably judge scene-relative object motion, even when heading errors for incorrect responses exceeded those for correct responses.

These findings strongly support the claim that scene-relative object motion judgment and heading estimation operate as separate processes and challenge the generality of the causal inference model in accounting for biases in heading estimation in the presence of independent object motion. Further research should aim to uncover the neural mechanisms driving these processes and develop new computational models that can capture the observed patterns of behavior.

## Supplementary Material

Supplement 1

Supplement 2

## Figures and Tables

**Figure A2.**
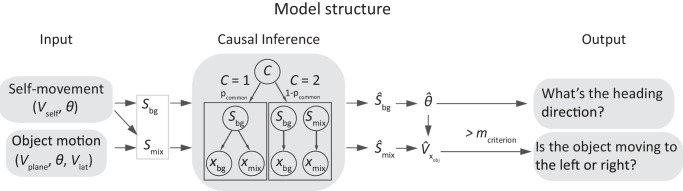
Bayesian causal inference model based on motion difference vector intersections. The model comprises three stages: input (left), causal inference (middle), and output (right). In the input stage, the model receives two types of signals: the observer's translational self-movement, defined by the speed *V_self_* and the heading direction (θ), and the object motion in the scene. The object motion in the scene is composed of two components: the motion of the plane with the same speed (denoted as *V_plane_* and equal to *V_self_*) and direction (θ) as the observer's translational self-movement (the plane moved in synchrony with the observer's forward translation, keeping the object dots on the plane at a constant distance of 1.58 m), and an additional = lateral motion of object dots on the plane (*V_lat_*). These inputs are used to compute the locations of the intersections of two types of motion difference vectors: one in the background optic flow (*S_bg_*), and one at the border of the moving object and the background optic flow (*S_mix_*). In the causal inference stage, we assume that *S_bg_* and *S_mix_* are drawn from Gaussian distributions with mean µ_p_ and standard deviation σ_p_, that is, *S_bg_* and *S_mix_* ∼ N(µ_p_, σ_p_^2^), and we set the mean of the prior distribution of *S_bg_* and *S_mix_* at the observer's straight-ahead direction, that is, µ_p_ = 0. Observers do not have access to the true intersections; therefore, *x_bg_* and *x_mix_* represent noisy measurements of *S_bg_* and *S_mix_*, respectively. These measurements are also drawn from Gaussian distributions, with *x_bg_* ∼ N(*S_bg_*, σ_bg_^2^) and *x_mix_* ∼ N(*S_mix_*, σ_mix_^2^). A Bayesian observer infers whether the sensory signals, *x_bg_* and *x_mix_*, originate from a common cause (C = 1) or from different causes (C = 2). For each scenario (C = 1 or C = 2), the posterior probability is calculated as the likelihood multiplied by its corresponding prior probability, where the prior probability of C = 1 is given by *p_common_* and that of C = 2 by 1 − *p_common_*. The observer then computes estimates of the locations of intersections under both scenarios (*C* = 1 and *C* = 2) and combines them with each estimate weighted by its posterior probability. In the output stage, the estimated intersections (${\hat{S}}_{bg}$ and ${\hat{S}}_{mix}$) are transformed into two task responses: the perceived heading direction ($\hat{\theta }$) and the perceived direction of the object's lateral motion in the scene ${\hat{V}}_{x_{obj}}=\;{\hat{V}}_{x_{plane}}\;+\;{\hat{V}}_{lat}$, where ${\hat{V}}_{x_{plane}}$ denotes the estimated lateral motion of the plane and ${\hat{V}}_{lat}$ denotes the estimated lateral motion of the object dots on the plane. The parameter *m_criterion_* represents a response-level bias that defines the threshold for perceiving rightward object motion.

**Figure A3.**
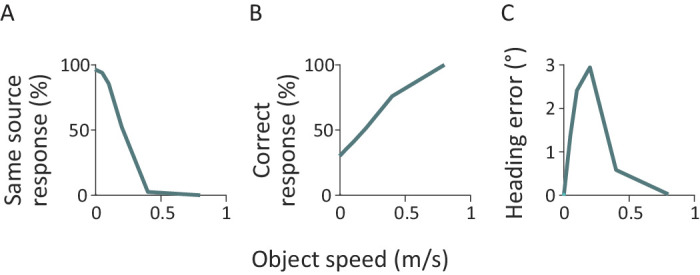
Causal inference model simulation results. (**A**) Same source response (%) and (**B**) correct response (%) of judging the object's lateral motion in the scene, and (**C**) heading error (°) as a function of object speed. To demonstrate the model's trends, we used the following parameter values: *p_common_* = 0.50, *σ_p_* = 1.5 (NDC), *σ_bg_* = 0.12 (NDC), *σ_mix_* = 0.17 (NDC), *m_criterion_* = 0.17 (m/s).

## Appendix

### Motion difference vectors

For heading perception in the presence of an independently moving object, there are two scenarios. One scenario is as tested in Dokka et al. (2019), that is, the moving object is approaching the observer while moving laterally in the scene. In this case, the heading bias is in the opposite direction of the object lateral motion (Koerfer & Lappe, 2020; Layton & Fajen, 2016a; Layton & Fajen, 2016b; Li et al., 2018; Warren & Saunders, 1995). The other scenario is what we tested in the current study, that is, the object moves laterally in the scene but does not simultaneously approach the observer. In this case, the heading bias is in the same direction of the object lateral motion (Koerfer & Lappe, 2020; Li et al., 2018; Royden & Hildreth, 1996; Royden & Hildreth, 1999). Given that the computational heading template model that simply pools all velocity vectors in the flow field for heading estimation (such as Warren & Saunders, 1995) could not explain this type of heading bias, Royden (2002) proposed a model based on motion difference vectors to account for the direction of heading biases caused by both approaching and non-approaching object motion.

Specifically, subtracting velocity vectors across adjacent points to obtain motion difference vectors in the optic flow field helps to reduce rotational noise in the flow field, because these difference vectors point directly toward the actual translational direction (Rieger & Lawton, 1985). When there are moving objects in the scene, motion difference vectors that naturally occur at its borders deviate from the motion difference vectors in the background optic flow associated with the stationary scene and thus the scene contains two FoEs (Figure A1). Royden (2002) has proposed that the center-surround neurons in the MT area can compute these motion difference vectors and feed them into the MST area that functions like heading templates. Royden further showed that, for a heading template model that pools the motion difference vectors for heading estimation, the bias in heading estimation is in the same direction of the object’s lateral motion, consistent with human data.

### Causal inference model

For the causal inference model in the current study, we adopted a key assumption grounded in Bayesian causal inference theory: the visual system would likely infer whether motion signals within the object region and the surrounding optic flow arise from a single common cause (self-movement) or from two distinct causes (self-movement plus object motion). We propose that when observers could reliably (i.e., ≥75% accuracy) judge the direction of object motion, they would likely attribute object and background motion to distinct sources and thus rely on the background optic flow for heading estimation, resulting in minimal heading bias. In contrast, when observers could not reliably judge object direction, they would likely treat the object motion as part of the global flow field, that is, arising from a single source, and thus produce a systematic bias in heading estimation using the entire flow field in the direction of object motion as shown by the difference vectors model (Royden, 2002). Our causal inference model consists of three parts: input stage, causal inference, and output stage, as shown in Figure A2.

### Input stage

In the input stage, the model receives two types of signals: the observer's translational self-movement in the scene, defined by the translational self-movement speed *V_self_* and heading (θ), and the object motion in the scene. The object motion in the scene is described as a combination of two components: a motion of the plane with the same speed (denoted as *V_plane_* and equal to *V_self_*) and direction (θ) as the observer's translational self-movement in the scene (the plane moved in synchrony with the observer's forward translation, keeping the dots on the plane at a constant distance of 1.58 m), and an additional object lateral motion on the plane (*V_lat_*). These inputs are used to compute the locations of the intersections of motion difference vectors.

The intersections of the difference vectors vary their locations in three distinct regions on the screen: the region containing only background dots, the region containing only object dots, and the border between the moving object and background optic flow where difference vectors can be computed by subtracting background from object motion vectors. The intersection of the motion difference vectors of the background dots, *S_bg_*, lies in the direction of the observer's self-movement in the scene (θ). The intersection of motion difference vectors of the object dots, *S_obj_*, lies in the direction of object motion relative to the observer. The intersection of motion difference vectors at the border of the moving object, *S_mix_*, lies in the direction of object motion relative to the background optic flow. Specifically, *S_mix_* will be the same as *S_obj_* if there is no self-movement, and *S_mix_* will be the same as *S_bg_* if there is no object motion.

Given that the motion difference vectors in the object region do not intersect in the current study (*S_obj_* is null), the model thus uses *S_bg_* and *S_mix_* as inputs. Because heading directions and object lateral motion on the plane were simulated only along the horizontal axis, *S_bg_* and *S_mix_* indicate horizontal positions of the intersections only for simplicity.

With screen coordinates normalized to [−1, 1] in both *x* and *y* coordinates (normalized device coordinates, NDC; left/down = negative, right/up = positive), *S_bg_* is given by:

(1) $$\begin{array}{c} S_{bg}=\;\frac{\tan\left(\theta \right)}{\tan\left(FoV_{x}\right)},\; \end{array}$$

where θ is the self-movement direction in degrees, and *FoV_x_* is the visual angle of the horizontal field of view in degrees.

*S_mix_* is given by:

(2) $$\begin{array}{ccc} & & \;S_{mix}=\;\frac{1}{\tan\left(FoV_{x}\right)d_{obj}}\\ & & \;\left(\left(\frac{d_{bg}}{\cos\left(\theta \right)V_{self}}-T_{\mathrm{diff}}\right)V_{lat}+d_{obj}\tan\left(\theta \right)\right),\; \end{array}$$

where *V_self_* denotes the speed of the simulated self-movement in the scene, *V_lat_* the speed of the object's lateral motion on the plane, *d_bg_* the average depth of the background dots, *d_obj_* the depth of the object dots on the plane, and *T_diff_* the time interval for estimating dot velocity on the display (each dot's velocity is computed as the difference between its screen positions at *t* = 0 and *t* = 0 + *T_diff_*, and we pick *T_diff_* = 1/60 second to estimate the velocity given our frame rate of 60 Hz).

### Causal inference stage

In the causal inference stage, we assume that *S_bg_* and *S_mix_* are drawn from Gaussian distributions with mean µ_p_ and standard deviation σ_p_, that is, *S_bg_* and *S_mix_* ∼ N(µ_p_, $\sigma_{p}^{2}$), and we set the mean of the prior distribution of *S_bg_* and *S_mix_* at the observer's straight-ahead direction, that is, µ_p_ = 0. Observers do not have access to the true intersections; therefore, *x_bg_* and *x_mix_* represent noisy measurements of *S_bg_* and *S_mix_*, respectively. These measurements are also drawn from Gaussian distributions, with *x_bg_* ∼ N(*S_bg_*, $\sigma_{bg}^{2}$) and *x_mix_* ∼ N(*S_mix_*, $\sigma_{mix}^{2}$).

A Bayesian observer faces the problem of determining whether the sensory signals *x_bg_* and *x_mix_* share a common source or not, and the task of estimating the direction of self-movement (θ) and the direction of the object's lateral motion in the scene ($V_{x_{obj}}$).

We assume two possible causal structures: a common source (*C* = 1) or independent sources (*C* = 2). The prior probability of *C* = 1 is denoted as *p_common_*, with the probability of *C* = 2 given by 1 − *p_common_*. Applying Bayes’ rule, the posterior probability that the horizontal location of the intersection of difference vectors at the object–background border (*S_mix_*) is the same as that in the background optic flow field (*S_bg_*) is given by:

(3) $$\begin{array}{ccc} & & \;P\left(C=1|x_{bg},x_{mix}\right)=\frac{P\left(x_{bg},x_{mix}|C=1\right)p_{common}}{P(x_{bg},x_{mix})}.\;\;\; \end{array}$$

Under the common-source assumption (*C* = 1), the maximum a posteriori probability estimate of *S_bg_* = *S_mix_* is a reliability-weighted (i.e., the inverse variance) average of sensory measurements *x_bg_* and *x_mix_*, as well as the prior mean *μ_p_*, which is given by:

(4) $$\begin{array}{c} {\hat{S}}_{bg,\;C=1}=\;{\hat{S}}_{mix,\;C=1}=\;\frac{\frac{x_{bg}}{\sigma_{bg}^{2}}+\frac{x_{mix}}{\sigma_{mix}^{2}}+\frac{\mu_{p}}{\sigma_{p}^{2}}}{\frac{1}{\sigma_{bg}^{2}}+\frac{1}{\sigma_{mix}^{2}}+\frac{1}{\sigma_{p}^{2}}}.\; \end{array}$$

The posterior probability that the intersection of motion difference vectors at the object–background border (*S_mix_*) is different from that in the background optic flow field (*S_bg_*) is given by:

(5) $$\begin{array}{ccc} & & \;P\left(C=2|x_{bg},\;x_{mix}\right)\\ & & \;=\;\frac{P\left(x_{bg},\;x_{mix}|C=2\right)\left(1-p_{common}\right)}{P\left(x_{bg},\;x_{mix}\right)}.\; \end{array}$$

Under the independent-source assumption (*C* = 2), the estimates of *S_bg_* and *S_mix_* are independent, with each computed as a reliability-weighted average of its corresponding sensory measurement (*x_bg_* or *x_mix_*) and the prior mean (*μ_p_*):

(6a) $$\begin{array}{c} {\hat{S}}_{bg,\;C=2}=\frac{\frac{x_{bg}}{\sigma_{bg}^{2}}+\frac{\mu_{p}}{\sigma_{p}^{2}}}{\frac{1}{\sigma_{bg}^{2}}+\frac{1}{\sigma_{p}^{2}}},\; \end{array}$$

(6b) $$\begin{array}{c} {\hat{S}}_{mix,\;C=2}=\frac{\frac{x_{mix}}{\sigma_{mix}^{2}}+\frac{\mu_{p}}{\sigma_{p}^{2}}}{\frac{1}{\sigma_{mix}^{2}}+\frac{1}{\sigma_{p}^{2}}}.\; \end{array}$$

Thus, ${\hat{S}}_{bg,\;C=1}$, ${\hat{S}}_{mix,\;C=1}$, ${\hat{S}}_{bg,\;C=2},$ and ${\hat{S}}_{mix,\;C=2}$ denote the estimated intersections under the assumptions of a common source (*C* = 1) or independent sources (C = 2), respectively. For the final estimates ${\hat{S}}_{bg}$ and ${\hat{S}}_{mix}$, we assume that the Bayesian observer combines the estimated intersections from the two scenarios weighted proportionally to their posterior probabilities (for more details, see the Bayesian generative model in Körding et al., 2007, which shares the same structure as our model).

(7a) $$\begin{array}{ccc} & & \;{\hat{S}}_{bg}={\hat{S}}_{bg,\;C=1}P\left(C=1|x_{mix},x_{bg}\right)\\ & & \;+\;{\hat{S}}_{bg,\;C=2}P\left(C=2|x_{mix},x_{bg}\right)\; \end{array}$$

(7b) $$\begin{array}{ccc} & & \;{\hat{S}}_{mix}={\hat{S}}_{mix,\;C=1}P\left(C=1|x_{mix},x_{bg}\right)\\ & & \;+{\hat{S}}_{mix,\;C=2}P\left(C=2|x_{mix},x_{bg}\right)\; \end{array}$$

### Output stage

In this stage, the estimated intersections ${\hat{S}}_{bg}$ and ${\hat{S}}_{mix}$ are converted into the perceived heading direction ($\hat{\theta }$) and the perceived direction (leftward or rightward) of the object's lateral motion in the scene (${\hat{V}}_{x_{obj}}=\;{\hat{V}}_{lat}\;+\;{\hat{V}}_{x_{plane}}$).

### Heading estimation

The estimated heading direction ($\hat{\theta }$) could be directly computed using the inverse function of (1):

(8) $$\begin{array}{c} \hat{\theta }=\mathrm{atan}\left(\tan\left(FoV_{x}\right){\hat{S}}_{bg}\right).\; \end{array}$$

### Object motion judgment

The object's lateral motion in the scene (*V_x_obj__*) is equal to the sum of the lateral motion of the plane (*V_x_plane__*) and the object dots' lateral motion on the plane (*V_lat_*).

The estimated lateral motion of the plane (${\hat{V}}_{x_{plane}}$) can be computed from the estimated heading direction ($\hat{\theta }$) and the self-movement speed (*V_self_*):

(9) $$\begin{array}{c} {\hat{V}}_{x_{plane}}=V_{plane}\sin\left(\hat{\theta }\right)=\;V_{self}\sin\left(\hat{\theta }\right).\; \end{array}$$

Using the inverse function of (Equation 2), we can get the estimate of the object dots' lateral motion on the plane (${\hat{V}}_{lat}$):

(10) $$\begin{array}{c} {\hat{V}}_{lat}=\;\frac{\tan\left(\frac{FoV_{x}}{2}\right)d_{obj}{\hat{S}}_{mix}-d_{obj}\tan\left(\hat{\theta }\right)}{\frac{d_{bg}}{\cos\left(\hat{\theta }\right)V_{self}}-T_{diff}}.\; \end{array}$$

The model then estimates the direction (leftward or rightward) of the object's lateral motion in the scene (${\hat{V}}_{x_{obj}}$) as:

(11) $$\begin{array}{c} {\hat{V}}_{x_{obj}}={\hat{V}}_{lat}+{\hat{V}}_{x_{plane}}.\; \end{array}$$

Because of flow parsing and induced motion, objects that are stationary but positioned to the right of the heading direction appear to move leftward. This perceptual bias implies that the response criterion is shifted rightward, rather than centered at zero. We refer to this shifted criterion as *m_criterion_*. Specifically, the model reports an object as moving rightward in the scene if ${\hat{V}}_{x_{obj}}\;>\;m_{criterion}$. From Equations (9) through (11), we derive the final inequality that specifies when to report rightward object motion in the scene:

(12) $$\begin{array}{ccc} & & \;\frac{\tan\left(\frac{FoV_{x}}{2}\right)d_{obj}{\hat{S}}_{mix}-d_{obj}\tan\left(\hat{\theta }\right)}{\frac{d_{bg}}{\cos\left(\hat{\theta }\right)V_{self}}-T_{diff}}\;\\ & & \;+\;V_{self}\sin\left(\hat{\theta }\right)\;>\;m_{criterion}.\; \end{array}$$

### Model simulations

Figure A3 shows the simulation results of our causal inference model for heading estimation with rightward object motion in the scene (averaged over 10,000 simulation runs). When object speed is relatively low, the motion difference vectors at the border of the moving object and those in the background flow are likely to be perceived as arising from a common cause. In this case, observers find it more difficult to judge the direction of object motion in the scene, and object motion is integrated into heading computation, leading to increased heading errors. As object speed continues to increase, the two types of motion difference vectors are more likely to be perceived as arising from distinct causes. In this case, observers can accurately judge the direction of object motion in the scene and estimate heading relying primarily on the background optic flow, resulting in a decrease in heading errors. Heading errors approaches zero when the accuracy of scene-relative object motion direction judgment reaches 100%.

### Pilot experiment: Determining experimental conditions

Before conducting the experiments, we ran a pilot experiment using the 1 m/s self-movement speed with a 2D display in Experiment 1 to examine the combinations of object positions relative to the FoE (both left and right) and object motion directions (see Figure A4).

Twenty students (6 males and 14 females; aged 18–28 years; mean±*SE*: 21.3 ± 2.93) from East China Normal University participated in the pilot experiment; all were naïve to the purpose of the study except one author. The testing procedure of the pilot experiment was identical to that of Experiment 1. The experiment had 220 experimental conditions (4 object position offsets × 11 object speeds × 5 heading directions) with 5 trials per condition, resulting in a total of 1,100 randomized trials. The entire session took approximately 80 minutes to complete.

The blue solid and dashed lines in Figure A4 represent conditions where the object motion direction matched its position relative to FoE (e.g., an object located on the right side of the FoE moving to the right), referred to as *same 8°* and *same 16°*. The red solid and dashed lines represent conditions where the object motion direction did not match its position relative to the FoE (e.g., an object located on the right side of the FoE moving to the left), referred to as *opposite 8°* and *opposite 16°*. From Figure A4, we can see that, for the same 8° and same 16° conditions, the correct response for object motion judgment increases with object speed much more slowly than for the opposite 8° and opposite 16° conditions where the background flow moved in the opposite direction as the object motion. For the latter two conditions, the correct response is suprathreshold (>80%), even at the samllest non-zero object speed of 0.05 m/s, making it difficult to evaluate the causal inference model. Accordingly, we decided to only test the same 8° and same 16° conditions in the real experiments.

### Control experiment: Testing eccentricity effect

We conducted a control experiment using the same display settings of the 1 m/s 2D display condition in Experiment 1 except that the background dots were stationary. Participants were asked to judge the object's motion direction as in Experiment 1 to examine whether the differences between the two position offsets persisted. Four students (2 males and 2 females; aged 26–34 years; mean, 28.75 ± 3.11) from East China Normal University participated in the control experiment; all were naïve to the purpose of the study. The control experiment included 120 conditions (4 object position offsets × 6 object speeds × 5 heading directions), with 5 trials per condition, resulting in a total of 600 randomized trials. The entire session took approximately 20 minutes to complete.

Figure A5 plots the percentage of correct response against object speeds. Except at zero object speed where the correct response rate was approximately 50–50, participants correctly identified the object motion direction 100% of the time for all other object speeds at both 8° and 16° object position offsets. This indicates that the differences in judging object motion direction in the scene for these two position offsets as observed in our experiments are unlikely due to eccentricity effect, but rather due to flow parsing.
